# Altered Functional Connectivity of Striatal Subregions in Patients with Multiple Sclerosis

**DOI:** 10.3389/fneur.2017.00129

**Published:** 2017-04-24

**Authors:** Fangyuan Cui, Li Zhou, Zengjian Wang, Courtney Lang, Joel Park, Zhongjian Tan, Yao Yu, Chunyan Sun, Ying Gao, Jian Kong

**Affiliations:** ^1^Department of Neurology, Dongzhimen Hospital, Beijing University of Chinese Medicine, Beijing, China; ^2^Department of Psychiatry, Massachusetts General Hospital, Harvard Medical School, Charlestown, MA, USA; ^3^Department of Radiology, Dongzhimen Hospital, Beijing University of Chinese Medicine, Beijing, China

**Keywords:** relapsing–remitting multiple sclerosis, resting-state functional connectivity, striatal subregions, dorsal caudal putamen, pathophysiology

## Abstract

Abnormal corticostriatal resting-state functional connectivity (rsFC) has been implicated in the neuropathology of multiple sclerosis. The striatum, a component of the basal ganglia, is involved in diverse functions including movement, cognition, emotion, and limbic information processing. However, the brain circuits of the striatal subregions contributing to the changes in rsFC in relapsing–remitting multiple sclerosis (RRMS) patients remain unknown. We used six subdivisions of the striatum in each hemisphere as seeds to investigate the rsFC of striatal subregions between RRMS patients and matched healthy controls (HCs). In addition, we also scanned a subcohort of RRMS patients after an average of 7 months to test the reliability of our findings. Compared to HCs, we found significantly increased dorsal caudal putamen (DCP) connectivity with the premotor area, dorsal lateral prefrontal cortex (DLPFC), insula, precuneus, and superior parietal lobule, and significantly increased connectivity between the superior ventral striatum and posterior cingulate cortex (PCC) in RRMS patients following both scans. Furthermore, we found significant associations between the Expanded Disability Status Scale and the rsFC of the left DCP with the DLPFC and parietal areas in RRMS patients. Our results suggest that the DCP may be a critical striatal subregion in the pathophysiology of RRMS.

## Introduction

The striatum is a component of the basal ganglia and is composed of the caudate nucleus, putamen, and nucleus accumbens. These regions are involved in sensorimotor function, cognition, and emotional information processing (1–3). Previous studies have demonstrated that the striatum interacts with other cortical regions to plan and execute goal-directed behavior (4–6), as well as motor activity (7). The striatum also receives projections from specific, remote cortical areas and projects them to the thalamus and brainstem *via* the pallidum and then back to the cortex. This pathway is known as the corticostriatal network (5).

Multiple sclerosis (MS) is characterized by multiple lesions in the brain and/or spinal cord, resulting in various nervous system impairments (8). In recent years, corticostriatal resting-state functional connectivity (rsFC) has been implicated in the pathophysiology of MS (9, 10). For example, using voxelwise analysis, Sbardella and colleagues found that MS patients showed reduced basal ganglia-related rsFC when compared with healthy controls (HCs) (11). In other studies, investigators found that MS patients had significantly reduced functional connectivity within the corticostriatal motor loop (12) and revealed a spatial expansion of motor resting-state connectivity in subcortical nuclei (13, 14). Furthermore, Finke et al. found that MS patients were associated with altered functional connectivity of the striatum with the frontal gyrus, prefrontal cortex, motor area, and parietal cortex (15).

Although abnormal corticostriatal functional connectivity has been detected in MS patients, the precise role of the striatal subdivisions in MS remains unclear. Individual striatal subregions are believed to connect to specific functional cortical networks (9, 16, 17). More specifically, the inferior ventral striatum is positively connected with the orbital frontal cortex and the arterial cingulate cortex, the dorsal caudate (DC) is positively connected with the superior and middle frontal gyri, and the dorsal caudal putamen is positively connected with the precentral gyrus.

Previous studies have also found that portions of the striatal subregions, such as the superior ventral striatum (VSs) and DC, demonstrate abnormal rsFC with reward-related brain regions in depression patients (18, 19). In children with autism, nearly all striatal subregions showed increased rsFC with the limbic cortex (20), and in patients with obsessive-compulsive disorder, there is a clear functional distinction in the corticostriatal axis between the dorsal and ventral striatal regions (21). Similarly, exploring rsFC changes in striatal subregions in MS patients may shed light on neural plasticity changes in MS patients.

In this study, we used six subdivisions of the striatum as seeds to investigate the rsFC of patients with relapsing–remitting multiple sclerosis (RRMS), as well as the association between rsFC and patients’ symptoms, as indicated by the Expanded Disability Status Scale (EDSS). We hypothesized that compared to HCs, RRMS patients would have altered corticostriatal functional connectivity of striatal subregions, particularly subregions of the putamen due to its important role in the corticostriatal motor loop. Additionally, in order to test the reliability of the corticostriatal rsFC changes in RRMS patients, we scanned a subcohort of the RRMS patients a second time in the fMRI scanner.

## Materials and Methods

### Participants

Twenty patients with a primary diagnosis of RRMS were recruited for this study at Dongzhimen Hospital, Beijing, China. All patients met the revised McDonald criteria (22) and classification standards for MS. Fifteen age and sex-matched right-handed healthy subjects were used as a control group. Healthy subjects had no history of neurological or psychiatric disease.

The inclusion criteria for RRMS patients were: (1) patients were in the remission stage of RRMS with no acute attack or exacerbation of MS during the last month; (2) patients were not taking any glucocorticoid medications; (3) patients’ medication and treatment had no significant recent adjustments; (4) patients had no history of serious psychiatric illness or neurological disease other than MS; (5) Chinese was their primary language; (6) patients were right-handed according to the modified Edinburgh Handedness Questionnaire (23); and (7) patients did not have any contraindications to MRI. Patients who had contraindications to MRI, poor quality MRI images, or showed one or more gadolinium-enhancing lesions during their baseline MRI were excluded.

A neurologist assessed patients’ disability using the EDSS (24) on the day of the neuropsychological assessment.

### fMRI Scan

Twenty RRMS patients (RRMS1 group) and 15 HCs received the first fMRI scan. In order to investigate the reliability of our findings, 12 RRMS patients received a second fMRI scan (RRMS2 group) approximately 7 months apart.

### fMRI Data Acquisition

In this study, all fMRI data and structural data were acquired using two-pulse sequences on a 3-T scanner (Siemens AG, Erlangen, Germany) with an 8-channel head coil. The functional data were collected using EPI sequences oriented parallel to the AC-PC line (repetition time: 2,000 ms, echo time: 30 ms, flip angle: 90°, matrix: 64 mm × 64 mm, field of view: 225 mm × 225 mm, slice thickness: 3.5 mm, 36 slices, no gap, 306 time points). A T1-weighted 3D magnetization-prepared rapid acquisition gradient echo sequence (repetition time: 2,700 ms, echo time: 2.97 ms, flip angle: 7°, matrix: 256 mm × 256 mm, field of view: 250 mm × 250 mm, slice thickness: 1 mm, 176 sagittal slices covering the whole brain, no gap, acquisition voxel size: 1 mm^3^) was also applied. Participants were instructed to lie still with their eyes closed while staying awake.

### fMRI Data Analysis

We examined six subregions of the striatum. These six subregions were widely used in previous studies (18, 20, 21). The six seeds included the inferior [Montreal Neurological Institute (MNI) peak coordinate *x y z*: ±9 9 −8] and superior (±10 15 0) ventral striata (inferior ventral striatum (VSi) and VSs), DC (±13 15 9), dorsal caudal putamen (DCP) (±28 1 3), dorsal rostral putamen (DRP) (±25 8 6), and ventral rostral putamen (VRP) (±20 12 −3) (16). Each striatal subregion was extracted as a 3 mm radial sphere using WFU-Pick Atlas software (25). The rsFC measures were computed between each seed and every voxel in the brain.

The preprocessing of fMRI data was performed with statistical parametric mapping (Wellcome Department of Cognitive Neurology, University College, London, UK) in MATLAB 8.2 (Mathworks, Inc., Natick, MA, USA). The preprocessing steps included realignment and coregistration of fMRI data to the structural images, normalization to the MNI standard template, and finally, smoothing of the data with an 8-mm full width at half maximum (FWHM) kernel. Band-pass filtering was performed with a frequency window of 0.008–0.09 Hz. In addition to our signals of interest, we employed segmentation of white matter and cerebrospinal fluid areas to remove confounding factors (26). Finally, time points were marked as outliers if the global mean intensity exceeded three SDs or if composite movement from a preceding image exceeded a 0.5 mm deviation using the artifact detection toolbox (http://www.nitrc.org/projects/artifact_detect/). Outlier time points were also included as regressors in the first-level general linear model along with motion parameters.

Functional connectivity analysis was carried out using a seed-based approach in the CONN toolbox v15.p (26). First-level correlation maps were produced by extracting the residual BOLD time course from each striatal seed region and by computing Pearson’s correlation coefficients between that time course and the time courses of whole-brain voxels. Correlation coefficients were Fisher transformed into “*Z*” scores, which increased normality and allowed for improved second-level general linear model analyses.

The second-level group analysis was applied using two-sample *t*-tests to compare the functional connectivity changes between the first scans of MS patients and HCs. Voxelwise linear regression analysis in CONN was also performed to investigate the association between the potential relationships between patients’ disability symptoms (EDSS) and the rsFC of each striatal subregion in the first and second scans of the MS patients. Age and gender were included in the analysis as covariates of non-interest.

A threshold of voxelwise *p* < 0.005 uncorrected and cluster-level *p* < 0.05 false discovery rate correction was applied for within and between-group fMRI data analyses.

### Behavioral Statistical Analysis

Behavioral analysis was performed using SPSS 18.0 Software (SPSS Inc., Chicago, IL, USA). Two-sample *t*-tests were applied to compare the baseline characteristics between the two RRMS patient cohorts and HCs.

## Results

### Patient Characteristics

Demographics and clinical characteristics are reported in Table 1. No significant differences were found between the RRMS1 patients and HCs in terms of age (*p* = 0.899) and gender (*p* = 0.835), and between the RRMS2 patients and HCs in terms of age (*p* = 0.960) and gender (*p* = 0.553). There were no significant differences (*p* = 0.903) between the first and second EDSS scores of the 12 RRMS2 patients.

**Table 1:** **Demographics and clinical characteristics of relapsing–remitting multiple sclerosis (RRMS) patients and healthy controls (HCs)**.

	HC, *n* = 15	RRMS1, *n* = 20	RRMS2, *n* = 12
Age (years)	36.7 (12.6)	37.2 (11.9)	36.4 (13.1)
Mean (SD)			

Gender	11/4	14/6	10/2
Female/male			

Expanded disability status scale score	NA	1.9 (1.4)	1.8 (1.6)
Mean (SD)			

### rsFC Results

**Figure 1:**
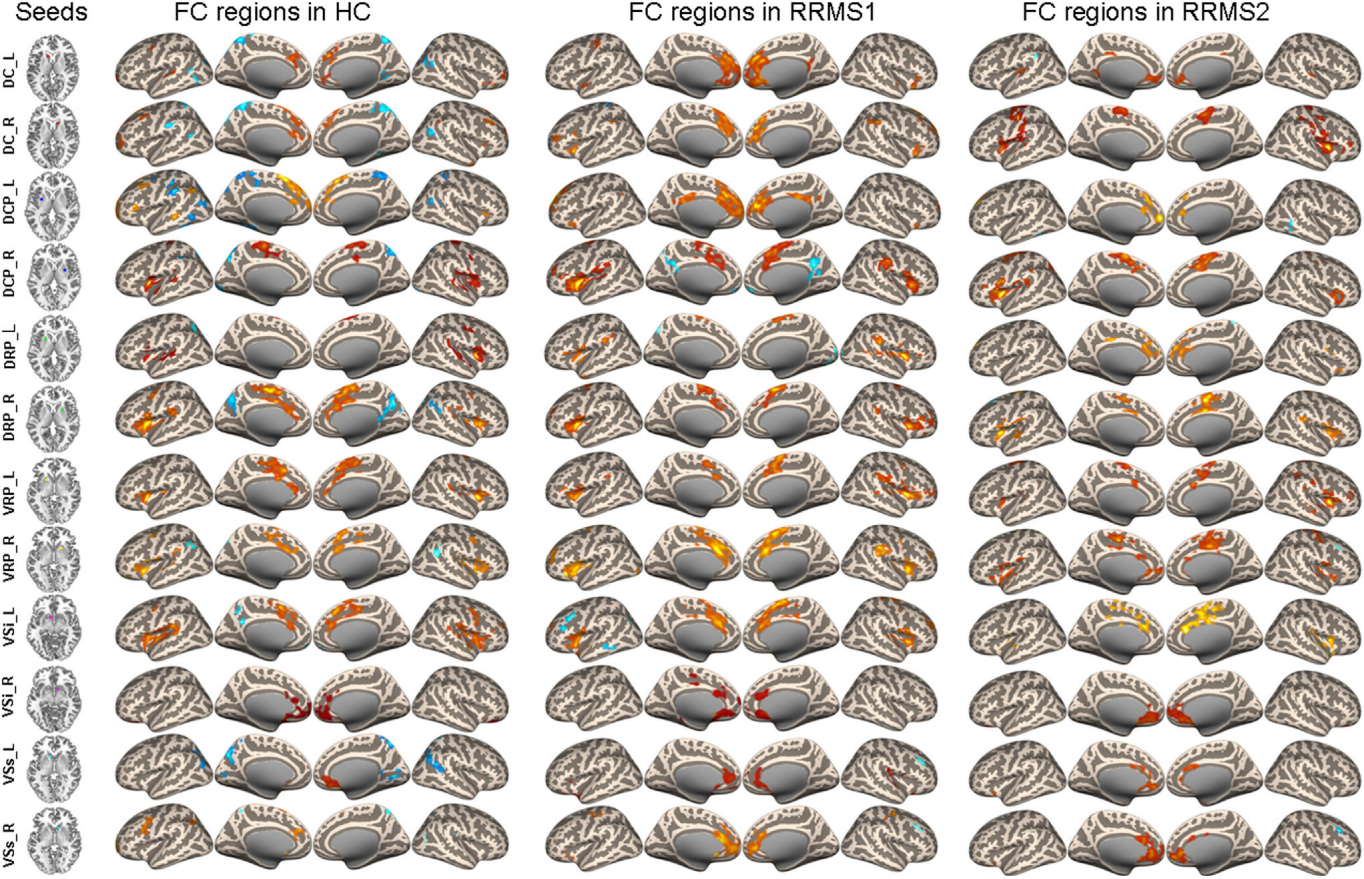
**Connectivity of caudate, putamen, and ventral striatum seeds in healthy controls (HCs) and RRMS1 and RRMS2 patients**.

A one-sample *t*-test was used to investigate rsFC patterns in RRMS patients and HCs (Figure 1). The results demonstrated that rsFC patterns in the RRMS patients and the controls were consistent with previous studies (9, 16).

### Comparison between RRMS Patients and HCs

#### Dorsal Caudal Putamen

**Figure 2:**
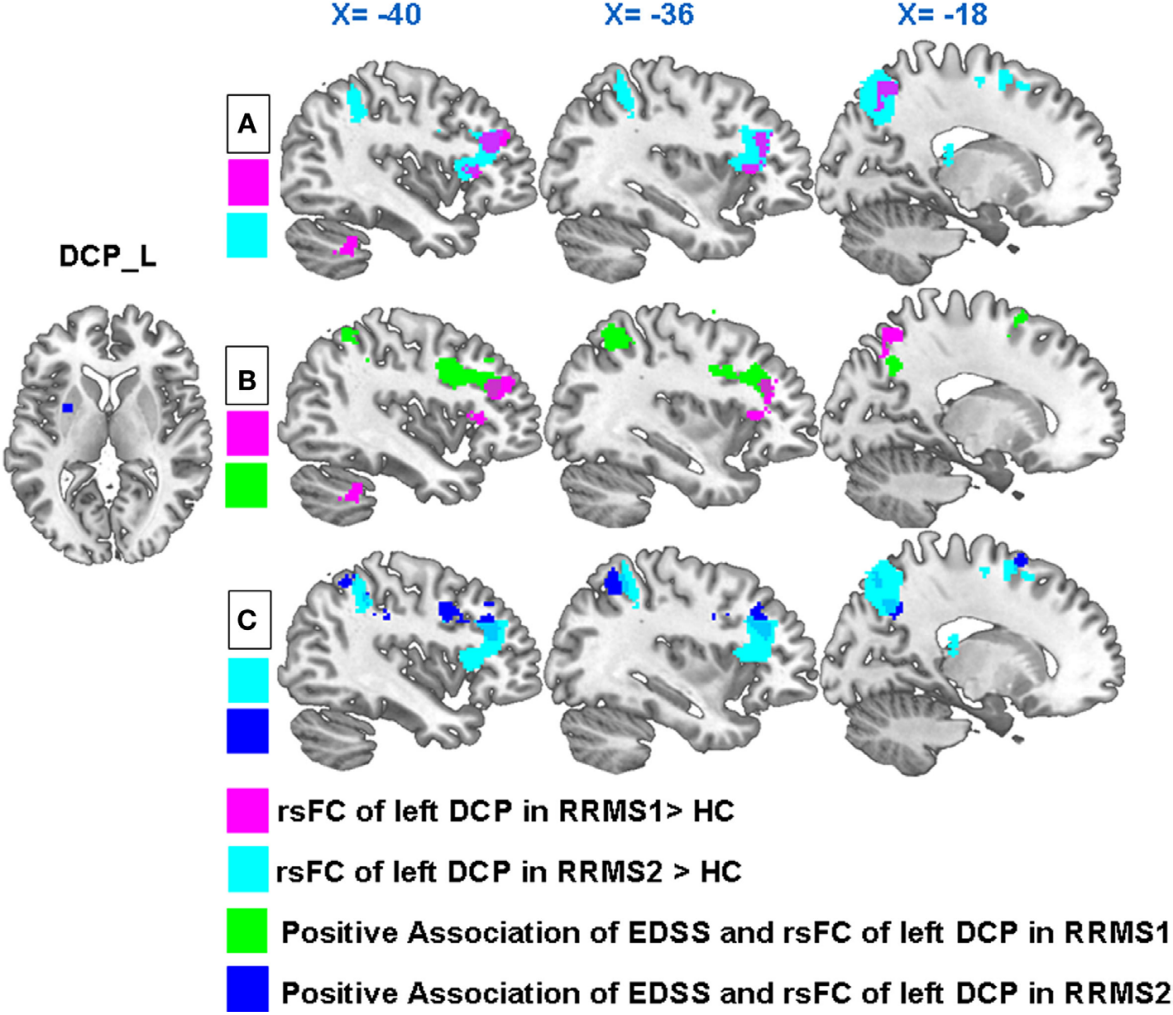
**(A)** In both RRMS1 and RRMS2 cohorts, the left dorsal caudal putamen (DCP) showed significantly increased FC with the left premotor cortex, dorsal lateral prefrontal cortex (DLPFC), insula, precuneus, and superior parietal lobule compared to healthy controls (HCs). **(B)** Overlapping regions of between-group comparisons and associations between the Expanded Disability Status Scale (EDSS) and left DCP FC at the DLPFC in the RRMS1 cohort. **(C)** Overlapping regions of between-group comparisons and associations between the EDSS and left DCP FC at the DLPFC and parietal areas in the RRMS2 cohort.

The left DCP region showed significantly increased rsFC with the right premotor cortex, left dorsal lateral prefrontal cortex (DLPFC), left insula, left precuneus, left superior parietal lobule, left cerebellum, right postcentral gyrus, and right inferior parietal lobule in RRMS1 patients (*n* = 20) compared with HCs (*n* = 15) (Table 2; Figure 2).

**Table 2:** **Brain regions showing significant striatal connectivity differences between relapsing–remitting multiple sclerosis (RRMS) patients and healthy controls (HCs)**.

Contrast	Seed	Connected regions	*X*, *Y*, *Z*	Cluster size	*Z*-score
HC > RRMS1	DC_L	Supplementary motor area (SMA), L	−38, 16, 46	213	2.95
		Cerebellum, R	44, −54, -48	477	4.43 3.85
	DC_R	Thalamus, R Putamen, R	16, −18, 2 28, -20, 0	278	3.98 3.51
	VRP_R	SMA, L Precentral, L	−54, 10, 42 −46, 0, 46	313	3.72 3.31
		Middle frontal gyrus, R	60, 24, 24	223	2.95
		Precental, R	42, 6, 34	259	3.53
		Premotor, L	−14, 14, 66	306	2.8
	VSs_L	Middle temporal, L	−52, −38, −12	321	3.03
		SMA, L	−54, 12, 42	263	2.96
	VSs_R	Orbital frontal cortex, R	46, 48, −2	282	3.32 3.22
RRMS1 > HC	DC_L	Paracentral lobule, L Precuneus Lateral parietal lobule	−10, −48, 78 −2, −50, 52 20, −46, 58	804	3.39 2.82 2.79
		Parahippocampal, L Cerebellum, L	−36, −36, −20 −40, −44, −24	255	4.29 3.37
		Middle temporal, R Occipital cortex, L	52, −64, 20 −40, −86, 18	268 247	3.5 3.96
	DCP_L	Premotor, R	26, 8, 66	168	3.67
		Dorsal lateral prefrontal cortex (DLPFC), L Insula, L	−40, 32, 22 −52, 40, 24	342	3.44 3.34
		Precuneus, L Superior parietal lobule, L	−16, −58, 52 −16, −70, 42	185	3.3 2.93
		Postcentral, R Inferior parietal lobule, R	58, −24, 36 58, −32, 40	185	3.38 3.31
		Cerebellum, L	−44, −48, 38	288	−4.33
	VSs_L	Middle temporal gyrus, R	52, −68, 10	439	4.08
	VSs_R	Cerebellum, R PCC, R	2, −46, 2 16, −56, 8	318	4.17 2.81
		Parahippocampal, L Thalamus, L	−24, −30, −6 −20, −30, 0	283	3.72 3.25
Overlap of RRMS1 > HC and RRMS2 > HC	DCP_L	Premotor, R	28, 4, 62	74	3.81
		DLPFC, L Insula, L	−40, 20, 10 −52, 40, 24 −30, 20, 12	257	4.38 3.27 3.48
		Precuneus, L Superior parietal lobule, L	−18, −68, 46 −12, −66, 56	171	5.12 4.61
	VSs_R	PCC, R	6, −54, 6	175	3.92

**Figure 3:**
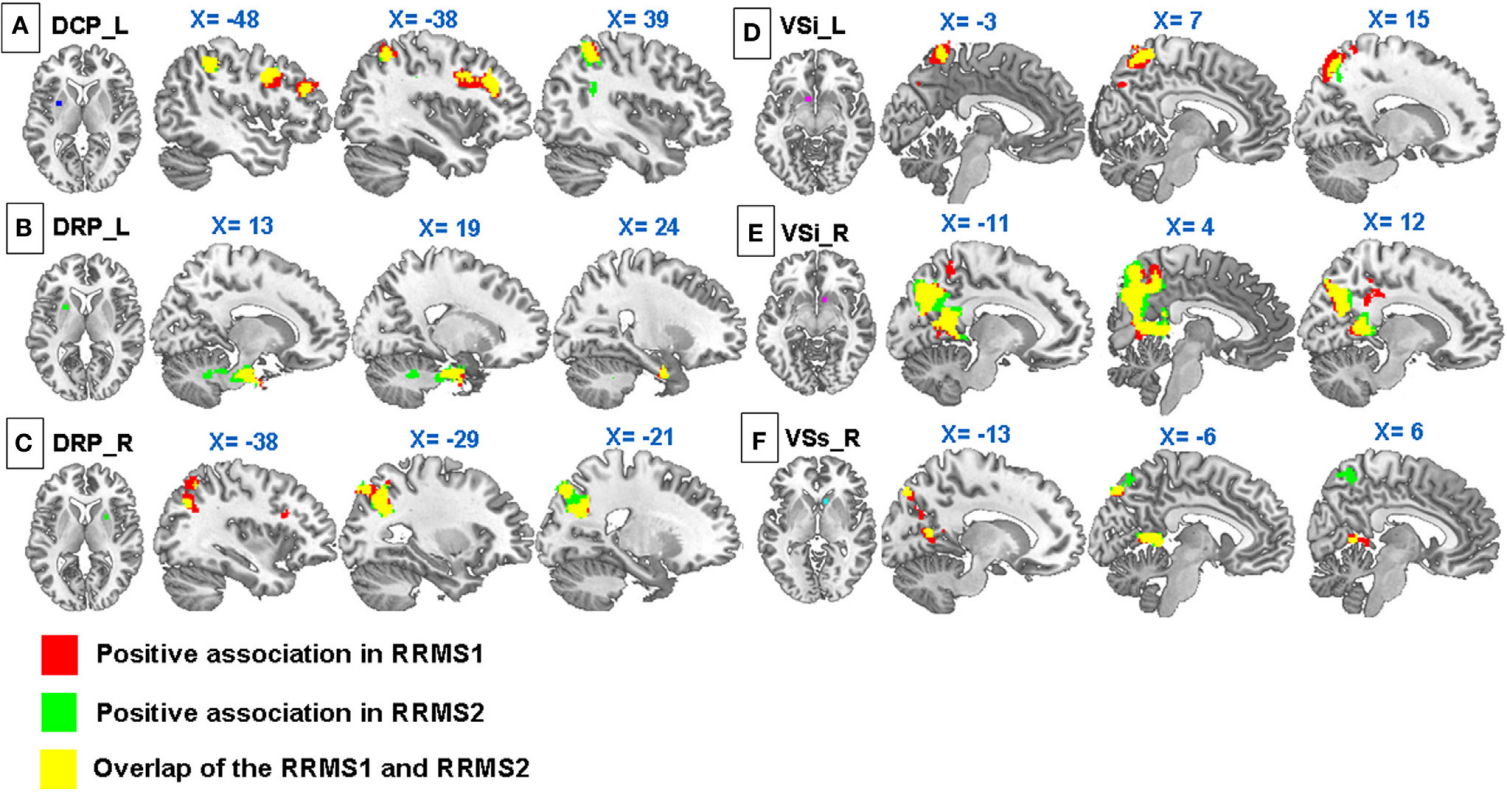
**Regression analyses showed significant positive associations between Expanded Disability Status Scale and (A) connectivity of the left DCP with the premotor cortex, DLPFC, inferior and superior parietal lobules, precuneus, and angular gyrus; (B) connectivity of the right dorsal rostral putamen (DRP) with the precuneus, superior parietal lobule, middle frontal gyrus, and precentral gyrus; (C) connectivity of the left DRP with the uncus and brainstem; (D) connectivity of the VSs with the precuneus, posterior cingulate cortex, and left occipital cortex; (E) connectivity of the left VSi with the precuneus, postcentral gyrus, superior parietal lobule, superior occipital gyrus, and middle temporal gyrus; (F) connectivity of the right VSi with the precuneus and PCC in RRMS1**. Similar associations were observed in RRMS2.

Regression analysis showed that there were significant positive associations between the EDSS scores and the rsFC of the left DCP with the left DLPFC, bilateral inferior parietal lobule, left superior parietal lobule, left precuneus, left premotor cortex, and right angular gyrus in RRMS1 patients (Table 3; Figure 3). Similar findings were also observed in the association analysis between EDSS scores and the rsFC of the left DCP in RRMS2 patients (Table 3; Figure 3).

**Table 3:** **Brain regions showing significant positive associations between Expanded Disability Status Scale scores and striatal connectivity in relapsing–remitting multiple sclerosis (RRMS) patients**.

Seeds	Associated regions	*X*, *Y*, *Z*	Cluster size	*Z*-score	*X*, *Y*, *Z*	Cluster size	*Z*-score
		RRMS1			RRMS2		
DCP_L	Dorsal lateral prefrontal cortex (DLPFC), L	−40, 30, 26 −34, 26, 32	960	4.9 4.01	−52, 8, 36 −38, 18, 32	348	3.55 3.53

	Superior parietal lobule, L Inferior parietal lobule, L Precuneous, L	−22, −60, 36 −34, −56, 52 −46, −42, 44	659	4.05 3.94 3.32	−22, −60, 38 −34, −54, 50 −42, −32, 34	892	4.28 4.05 3.22

	Premotor, L	−22, 10, 66 −20, 2, 54	295	3.85 3.09	−22, 10, 66 −28, 10, 70	208	4.02 3.57

	Inferior parietal lobule, R Angular, R	42, −52, 58 38, −50, 54	280	3.67 3.63	42, −52, 58 38, −50, 54	494	3.65 3.61

	Supplementary motor area, L	–	–	–	−38, 30, 24 −40, 32, 40	245	4.27 2.68

	DLPFC, R	–	–	–	44, 34, 36 38, 36, 34	184	3.96 3.93

DRP_L	Uncus, R Brainstem, R	18, −10, −32 22, −8, −30	288	3.90 3.69	14, −24, −32 18, −10, −32	629	4.67 3.43

DRP_R	Precuneus, L Superior parietal lobule, L	−20, −68, 28 −34, −66, 34	960	4.27 3.82	−20, −68, 28 −28, −64, 44	958	4.07 3.23

	Middle frontal gyrus, L Precentral, L	−46, 14, 34 −54, 0, 44	308	3.39 3.26	–	–	–

VSs_R	PCC, R/L Occipital cortex, L Precuneus, R/L	−18, −64, 14 10, −56, 2 −22, −64, 16 −10, −62, 8	1,014	3.84 3.83 3.8 3.58	−14, −64, 12 8, −56, 6 6, −58, 58	783	3.55 3.18 3.25

VSi_L	Precuneus, R/L Postcentral gyrus, L	22, −46, 68 −6, −72, 48 46, −70, 36	1,066	3.32 2.82 3.26	12, −66, 46 12, −74, 50	456	2.92 2.84

	Superior occipital gyrus, L Middle temporal gyrus, L	−40, −64, 24 −36, −82, 34	277	3.60 3.45	–	–	–

	Superior parietal lobule, L Precuneus, L	−16, −58, 46 −16, −62, 58	273	3.38 3.30	–	–	–

VSi_R	Precuneus, R/L PCC, R/L	−16, −74, 34 12, −68, 28 6, −52, 8	5,732	4.09 4.08 3.91	−14, −74, 28 6, −52, 8 −16, −74, 34	6,019	4.10 4.03 3.93

Interestingly, there were overlapping regions at the DLPFC and parietal areas for both group comparisons and regression analyses (Figures 2B,C).

#### Dorsal Rostral Putamen

There was no significant difference in DRP rsFC between the RRMS1 patients and HCs.

**Figure 4:**
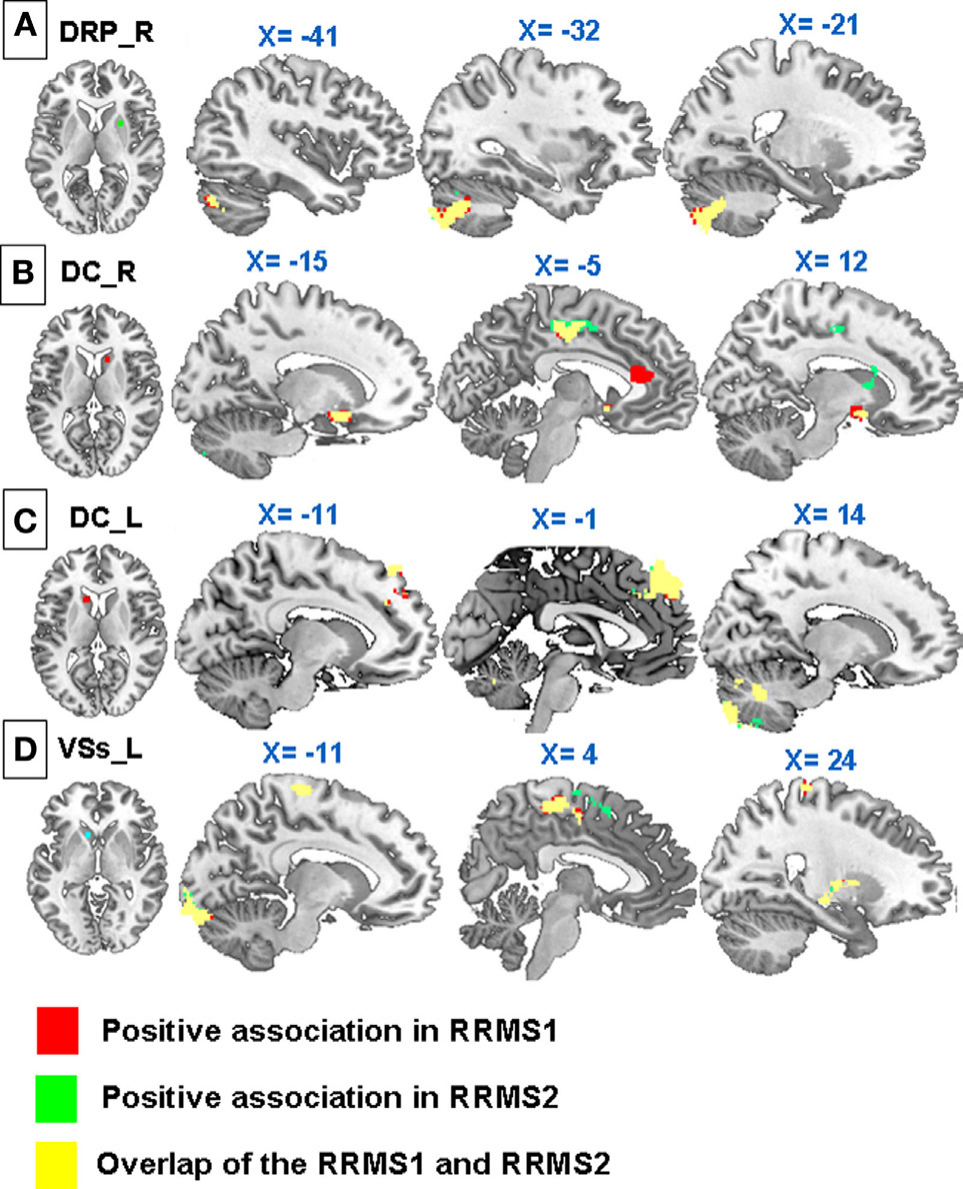
**Regression analyses showed significant negative associations between Expanded Disability Status Scale scores and (A) connectivity of the dorsal rostral putamen (DRP) with the bilateral anterior cingulate cortex, premotor area, left amydala, and paracentral lobule; (B) connectivity of right dorsal caudate (DC) with the cerebellum; (C) connectivity of the left DC with the supplementary motor area, premotor cortex, and cerebellum; (D) connectivity of the left VSs with the premotor cortex, insula, pallidus, putamen, thalamus, lingual gyrus, and occipital lobe in RRMS1**. Similar associations were observed in RRMS2.

Regression analyses showed that there were significant positive associations between the EDSS scores and the rsFC of the right DRP with the left precuneus, left superior parietal lobule, left middle frontal gyrus, and left precentral gyrus, and the left DRP with the right uncus and right brainstem in the RRMS1 patients (Table 3; Figure 3). There was also a negative association between EDSS scores and the rsFC of the right DRP with the bilateral anterior cingulate cortex, premotor area, left amygdala, and paracentral lobule (Table 4; Figure 4) in RRMS1 patients. Similar findings were also observed in RRMS2 patients (Tables 3 and 4; Figures 3 and 4).

**Table 4:** **Brain regions showing significant negative associations between Expanded Disability Status Scale scores and striatal connectivity in relapsing–remitting multiple sclerosis (RRMS) patients**.

Seeds	Associated regions	*X, Y, Z*	Cluster size	*Z*-score	*X*, *Y*, *Z*	Cluster size	*Z*-score
		RRMS1			RRMS2		
DC_L	Cerebellum, R	16, −64, −32 14, −84, −46	992	4.56 4.53	16, −64, −32 6, −70, −24	1,027	4.66 3.56

	SMA, L Premotor, L	−4, 42, 50 −4, 38, 36	670	3.26 2.97	−4, −46, 44 −4, 38, 36	632	3.4 3.01

DC_R	Cerebellum, R	−24, −68, −38	792	4.34	−24, −66, −36	712	4.14

DRP_R	ACC, R/L Amygdala, L	4, 10, −8 −16, 4, −14 −22, −2, −14	448	3.76 3.18 3.09	−18, 10, −14 2, 10, −6 −24, −2, −14	299	3.46 3.22 2.85

	Paracentral lobule, L Premotor, L/R	−6, −20, 46 8, −12, 48 −6, −12, 50	243	3.65 3.22 3.21	8, −10, 50 −8, −24, 50 −2, −14, 46	559	3.92 3.75 3.39

VSS_L	Premotor, R	6, −10, 50	770	2.87	6, −10, 46	824	2.9

	Insula, R Pallidus, R Thalamus, R Putamen, R	38, −6, 14 32, −4, −2 26, −10, 0 16, −14, 6	436	3.81 3.66 3.57 3.56	38, −6, 14 26, −10, 0 16, −14, 6 28, 0, 2	466	3.91 3.61 3.59 3.40

	Lingual gyrus, L Occipital lobe, L	−22, −106, −10 −10, −100, −6	513	3.61 2.95	−20, −96, −12 −10, −100, −6	559	3.53 3.01

#### Ventral Rostral Putamen

There was decreased rsFC of the VRP with the left premotor area, bilateral precentral gyrus, left supplementary motor area (SMA), and right DLPFC in RRMS patients compared to HCs (Table 2).

Regression analyses showed there were no significant associations between EDSS scores and rsFC in both RRMS1 and RRMS2 patients.

#### Dorsal Caudate

RRMS patients showed significantly reduced rsFC of the left DC with the left SMA and right cerebellum, and the right DC with the right thalamus and right putamen compared to HCs. Additionally, RRMS patients showed increased rsFC between the left DC and the left paracentral lobule, left precuneus, right lateral parietal lobule, left parahippocampal gyrus, left cerebellum, left occipital cortex, and right middle temporal gyrus compared to HCs (Table 2).

Regression analyses showed negative associations between EDSS scores and the functional connectivity of the bilateral DC with the right cerebellum and left DC with the left SMA and premotor area in both RRMS1 and RRMS2 patients (Table 4; Figure 4).

#### Superior Ventral Striatum (VSs)

**Figure 5:**
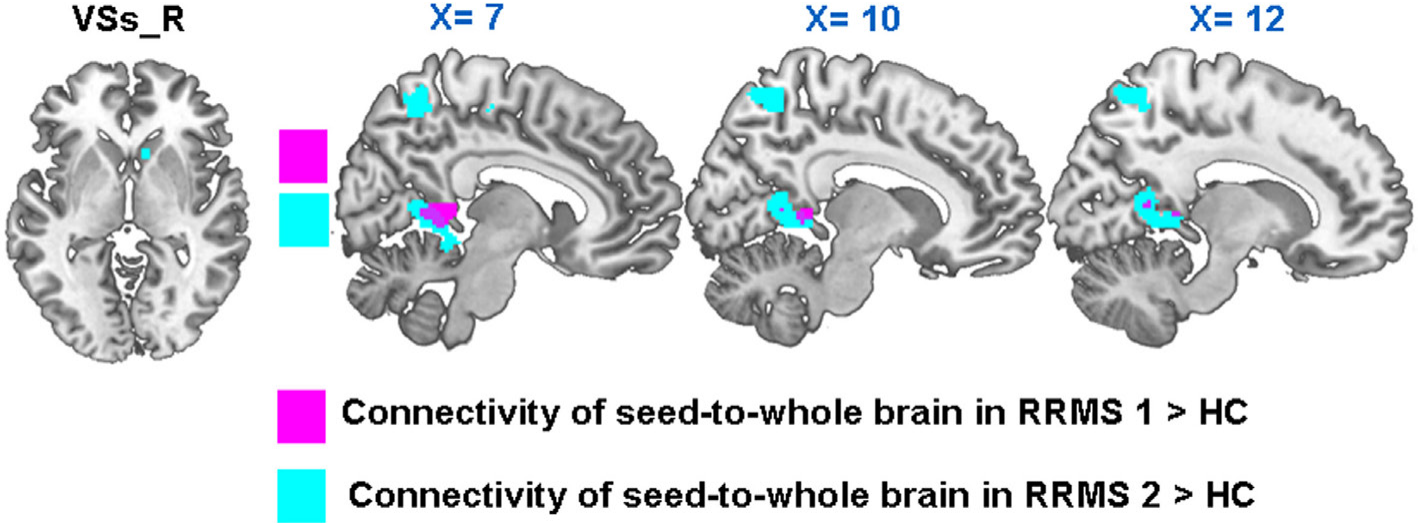
**In both RRMS1 and RRMS2, the right VSs had significantly increased connectivity with the right PCC compared to healthy controls (HCs)**.

Compared with HCs, RRMS1 patients showed increased rsFC between the right VSs and the right posterior cingulate cortex (PCC) and right cerebellum; increased rsFC between the left VSs and the right middle temporal gyrus; decreased rsFC between the left VSs and the left middle temporal gyrus, premotor cortex and left SMA; and decreased rsFC between the right VSs and the right orbital frontal cortex (Table 2; Figure 5).

Regression analyses showed that there were significantly positive associations between EDSS scores and the rsFC of the right VSs with the bilateral precuneus, posterior cingulate cortex, and left occipital cortex (Table 3; Figure 3), and negative associations between EDSS scores and the rsFC of the left VSs with the right premotor area, insula, pallidus, putamen, thalamus, left lingual gyrus, and occipital lobe in RRMS1 patients (Table 4; Figure 4). Similar findings were also observed RRMS2 patients (Tables 3 and 4; Figures 3 and 4).

#### Inferior Ventral Striatum (VSi)

There were no significant differences in the rsFC of the VSi between RRMS patients and HCs.

Regression analyses showed a strong positive association between EDSS scores and the rsFC of the VSi with the precuneus, posterior cingulate cortex, left postcentral gyrus, left parietal lobule, and superior parietal lobule (Table 3; Figure 3). Similar findings were also observed in RRMS2 patients (Table 3; Figure 3).

**Figure 6:**
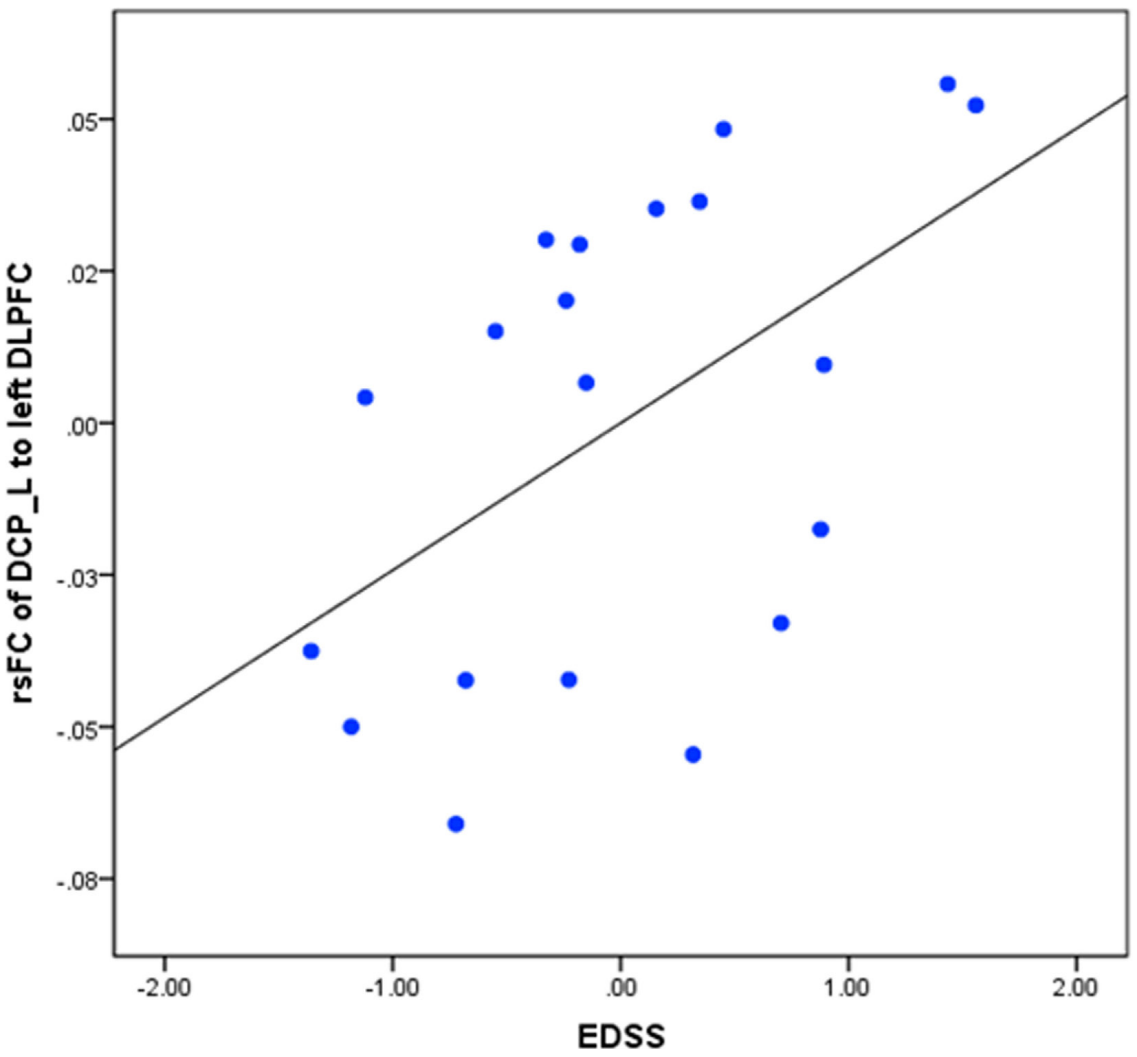
**Regression analysis between the resting-state functional connectivity (rsFC) of the left dorsal caudal putamen (DCP) and left dorsal lateral prefrontal cortex (DLPFC) in RRMS1 patients**.

We also explored the association between rsFC and EDSS scores between RRMS1 patients and HCs. First, we extracted and designated the significantly changed clusters between the RRMS1 patients and HCs as the ROIs, and then extracted the “*Z*” scores of each RRMS1 cluster. Finally, we performed a regression analysis to explore the association between the EDSS scores and “*Z*” scores of each RRMS1 cluster. Age and gender were included as covariates of non-interest. The results showed that there was a significant positive correlation between the EDSS scores and the connectivity of the left DCP with the left DLPFC (*p* = 0.031), which is a region of overlap between RRMS1 and RRMS2 patients and HCs (Figure 6).

### Overlap Regions between RRMS1 and RRMS2 Patients and HCs

**Figure 7:**
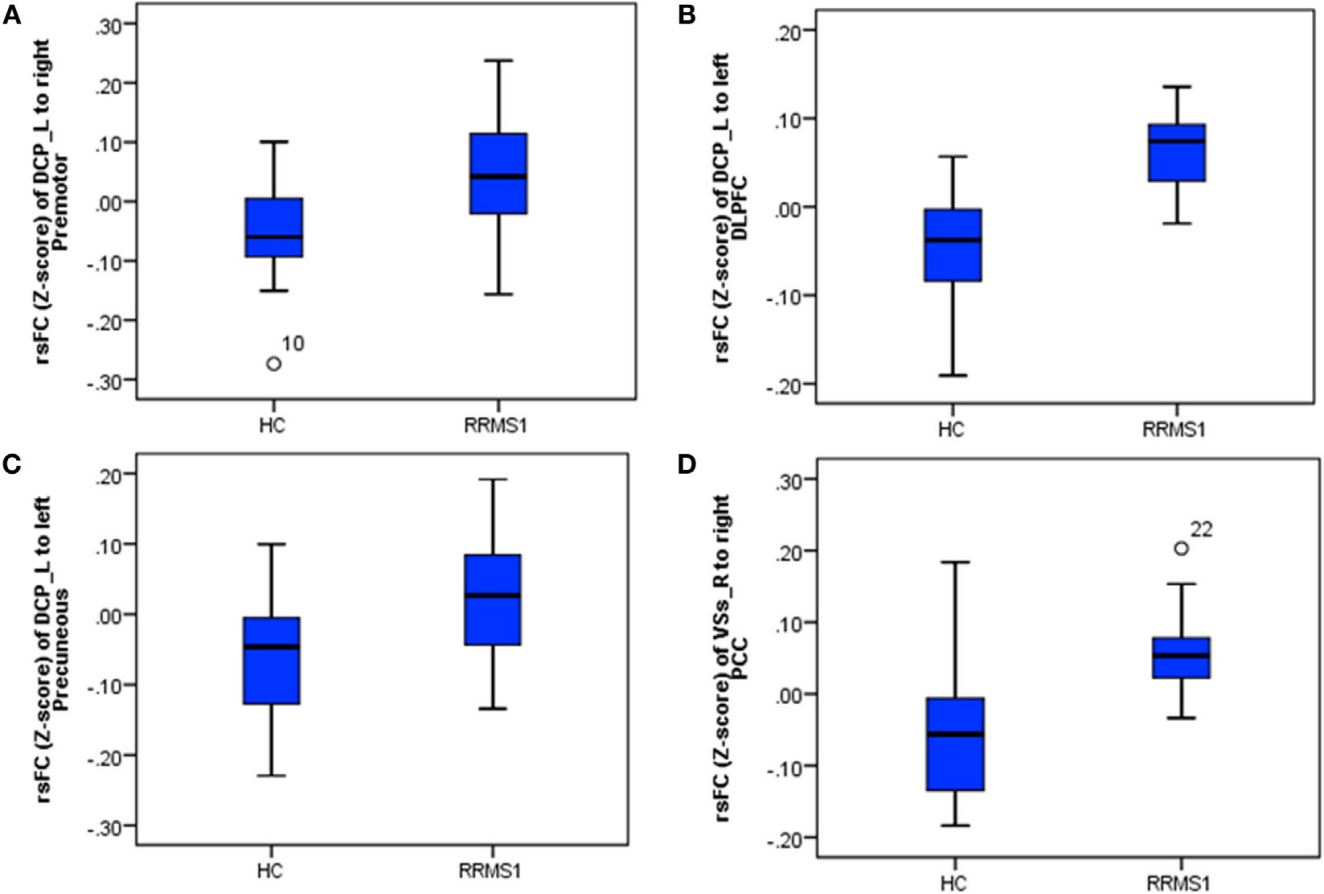
**The connectivity *Z*-scores of the main findings in RRMS1 patients and healthy controls (HCs), which were overlapped in the findings of the comparison between RRMS2 and HCs. (A–C)** *Z*-scores of connectivity between the left dorsal caudal putamen (DCP) and right premotor area/left dorsal lateral prefrontal cortex (DLPFC)/left precuneus in HC and RRM1 groups. **(D)** *Z*-scores of connectivity between the right ventral striatum (VSs) and right posterior cingulate cortex (PCC) in HC and RRMS1 groups.

Twelve RRMS patients were scanned twice and then compared with HCs. We found significantly increased connectivity in both comparisons including (1) increased rsFC of the left DCP with the left premotor cortex, DLPFC, insula, precuneus, and superior parietal lobule (Table 2; Figures 2A and 7) and (2) increased rsFC between the right VSs and the right posterior cingulate gyrus (Table 2; Figure 7).

### Comparison between the First and Second Scans of the 12 RRMS2 Patients

In the second scan, RRMS patients showed increased rsFC between (1) the left DCP and the left thalamus; (2) the left VRP and the right orbital frontal cortex and left paracingulate gyrus; (3) the right VRP and the right cerebellum; (4) the left VSi and the left orbital frontal cortex; and (5) the right DRP and the right precentral gyrus and angular and superior parietal lobules (Table 5).

**Table 5:** **Brain regions showing significant differences between the first and second scans in 12 relapsing–remitting multiple sclerosis (RRMS) patients**.

Contrast	Seed	Connected regions	*X*, *Y*, *Z*	Cluster size	*Z*-score
RRMS2 (*n* = 12) > RRMS1 (*n* = 12)	DCP_L	Thalamus, L	−12, −22, 14	292	3.66
	VRP_L	Orbital frontal cortex, R Paracingulate gyrus, L	6, −26, 18 −10, 32, −20	808	4.26
	VRP_R	Cerebellum, R	42, −46, −32	438	4.12
	DRP_R	Precentral, R	14, −16, 76	227	4.04
		Angular, R Superior parietal lobule, R	38, −38, 36 40, −48, 42	290	4.02
	VSi_L	Orbital frontal cortex, L	−20, 32, 4	445	4.57

## Discussion

In this study, we used seed-based resting-state fMRI to explore the corticostriatal circuitry in RRMS patients and the association between symptoms and functional connectivity. We found enhanced rsFC between the left DCP and the left premotor cortex, left DLPFC, insula, and parietal areas, and between the right VSs and the right posterior cingulate cortex in RRMS patients compared to HCs. We also found a positive association between corticostriatal rsFC and EDSS scores in RRMS patients. Specifically, after comparing RRMS1 patients with HCs and performing a regression between clinical outcomes and connectivity, we found overlapping results at the DLPFC and parietal areas when using the DCP as a seed. This result highlights the importance of the DCP in MS. In addition, a subcohort of RRMS patients was scanned twice in order to test the reliability of our findings. We found similar results after comparing both scans. Our results demonstrate the involvement of the striatum, particularly the DCP, in the pathophysiology of RRMS.

Previous studies have indicated that extensive lesions (8) might be the cause of the dysregulation of movement and the reduced connectivity of the motor network in the corticalsubcortical network in RRMS patients (13, 14, 27, 28). The basal ganglia interacts with other cortical regions to play a key role in contextually based motor decision making (7). More specifically, the striatum is thought to play a critical role in the storage and retention of motor programs (29). Within the striatum, the dorsal lateral putamen receives projections from the motor and motor association cortices (5, 30, 31), the main domain connected to the putamen seeds in healthy subjects (9, 16). Previous studies have found that the putamen is also connected to the DLPFC (17, 32–34) and the cingulate cortex (35), which implies that it is also involved in cognition and emotion.

In this study, we found increased rsFC between the DCP and the premotor cortex, insula, DLPFC, precuneous, and superior parietal lobule in both RRMS1 and RRMS2 patients when compared to HCs. We also found increased rsFC of the DCP with the DLPFC and parietal cortex. This increase was positively associated with EDSS scores in RRMS patients.

The test-retest findings indicate the important role of the rsFC of the DCP with the DLPFC and parietal gyrus in the pathophysiology of RRMS. The results are consistent with findings from previous studies indicating the involvement of the striatum in MS (13–15, 36). The DLPFC has diffuse connectivity with the rostral and caudal components of both the putamen and caudate, which project extensively to the central striatum and bridges the ventral and dorsal areas of the striatum (1). Studies suggest that the DLPFC plays an important role in motor and sensory information processing (37, 38), maintenance of sensory stimuli and motor execution (37), and pre-movement processes (39). Furthermore, the DLPFC is characterized as a part of the executive control network, which monitors motor performance (40) and integrates motor information and sensorimotor transformations (41).

The executive control network interacts with memory, motor, and sensory structures to direct thought and action (42). Dysfunction of the prefrontal cortex has been shown to cause deficits in executive function (43), and in studies of stroke patients, disruption of the executive control network has been shown to result in motor response deficits (44). We speculate that enhanced rsFC between the DCP and DLPFC may result in enhanced monitoring of motor performance (40) and enhanced integration of information and sensorimotor transformations (41).

Compared to HCs, RRMS patients showed increased rsFC of the left DCP with the parietal cortex, including the left precuneus, left superior parietal lobule, and right inferior parietal lobule. The superior parietal lobe is associated with the ability to produce purposeful and skilled movements and participates in complex motor programming. Studies have shown that motor execution requires coherent representations of neural composition and that the movement of a limb may cause activity changes in the left posterior parietal cortex (45). Previous studies found that, during the execution phase, patients with brain injuries showed enhanced activation in the superior and inferior parietal lobes, precuneus, and DLPFC compared with HCs. The superior parietal lobule and DLPFC, key regions in the frontoparietal control network, are known to have reciprocal circuit connectivity (46, 47) and play a role in changing spatial coordinates and body posture (45). It was also found that the functional connectivity of the parietal and frontal cortex can be altered by motor learning (48). Studies suggest that functional plasticity may occur following motor rehabilitation in patients with MS (49), and disrupted rsFC has been associated with disability progression in MS patients (50).

Previous studies showed that MS patients may need to activate more widespread sensorimotor networks compared to HCs during a sequential finger-to-thumb opposition task, as well as in other motor-related tasks (51, 52). Considering the dysfunction in movement, sensation, and cognition in RRMS (8), the increased rsFC of the dorsal putamen with the motor associated cortex and the positive association with clinical disability suggests that there is a compensatory modulation of the movement system in response to axonal injury (49). Taken together with these findings, our results suggest that the increased rsFC of the dorsal putamen with the DLPFC and parietal areas in RRMS patients may signal a compensatory connection for maintaining the function of the executive network or may adjust information resources for the motor processes involved in cognitive function.

We also found increased rsFC of the right VSs with the right PCC in RRMS1 and RRMS2 patients compared to HCs. The PCC is one of the primary nodes of the default mode network (DMN) (53, 54) and is implicated in memory processing and emotion generation (55). In a previous study, we found increased rsFC between the ventral striatum and the DMN in subthreshold depression patients compared with HCs, which may reflect a self-compensation mechanism to ameliorate the patient’s lower reward function (56). The PCC is shared between both the DMN and the sensorimotor network and, therefore, serves as an important interaction hub (57). The rsFC between the bilateral PCC and the ACC was increased following cognition and motor recovery after treatment in stroke patients (58). The PCC was also identified as the area involved in preparatory motor execution during rest (59) and directing the segregation of motor input (4). We thus speculate that the increased rsFC of the ventral striatum in RRMS patients may be due to the multiple neurological impairments of the motor and cognition system and may represent a complex modulatory interaction of the corticostriatal networks.

Finally, RRMS2 patients showed increased rsFC between the putamen and the primary motor cortex, thalamus, cerebellum, orbital frontal cortex, and parietal area compared with RRMS1 patients. The increased rsFC of the putamen confirmed the prior findings that displayed the functional coupling of the dorsal putamen and motor cerebral networks (9, 16). Previous rs-fMRI studies suggest the presence of functional plasticity following motor rehabilitation in MS (60). Based on this previous research, we speculate that the increased rsFC of the dorsal putamen with the motor cortex may be due to cortical reorganization accompanying the progression of RRMS.

One limitation of our study is the difference in lesion location among RRMS patients. MS lesions can be widespread across the brain and vary from patient to patient. We acknowledge that different lesion distributions may alter rsFC differently. Further studies with larger sample sizes are needed to further validate our findings.

## Conclusion

We observed a number of intriguing and significant changes in rsFC in striatal subregions between RRMS patients and HCs. Specifically, we found that the dorsal caudal putamen demonstrated increased and reliable rsFC with the DLPFC and parietal areas in RRMS patients. These rsFC results were significantly associated with the clinical symptoms. Our results demonstrate that the dorsal caudal putamen may be a key striatal subregion in the pathophysiology of RRMS.

## Ethics Statement

This study was approved by and carried out in accordance with the medical ethics committee at Dongzhimen Hospital, Beijing University of Chinese Medicine. All subjects gave written informed consent in accordance with the Declaration of Helsinki.

## Author Contributions

Experimental design: FC, LZ, YG, and JK; data collection: FC, LZ, ZT, YY, and CS; data analysis: FC, ZW, and JK; manuscript preparation: FC, LZ, JK, ZW, CL, and JP.

## Conflict of Interest Statement

The authors declare that the research was conducted in the absence of any commercial or financial relationships that could be construed as a potential conflict of interest.

## References

[1] Draganski B Kherif F Klöppel S Cook PA Alexander DC Parker GJM Evidence for segregated and integrative connectivity patterns in the human basal ganglia. J Neurosci (2008) 28(28):7143–52. 10.1523/JNEUROSCI.1486-08.2008 18614684 PMC6670486

[2] Middleton FA Strick PL Basal-ganglia “projections” to the prefrontal cortex of the primate. Cereb Cortex (2002) 12(2):926–35. 10.1093/cercor/12.9.926 12183392

[3] Pessiglione M Seymour B Flandin G Dolan RJ Frith CD. Dopamine-dependent prediction errors underpin reward-seeking behaviour in humans. Nature (2006) 442(7106):1042–5. 10.1038/nature05051 16929307 PMC2636869

[4] Arrubla J Tse DHY Amkreutz C Neuner I Shah NJ. GABA concentration in posterior cingulate cortex predicts putamen response during resting state fMRI. PLoS One (2014) 9(9):e106609. 10.1371/journal.pone.0106609 25184505 PMC4153676

[5] Haber SN. The primate basal ganglia: parallel and integrative networks. J Chem Neuroanat (2003) 26(4):317–30. 10.1016/j.jchemneu.2003.10.003 14729134

[6] Haber SN Corticostriatal circuitry. Dialogues Clin Neurosci (2016) 18(1):7–21. 10.1007/978-1-4614-6434-1_135-1 27069376 PMC4826773

[7] Tunik E Houk JC Grafton ST Basal ganglia contribution to the initiation of corrective submovements. Neuroimage (2009) 47:1757–66. 10.1016/j.neuroimage.2009.04.077 19422921 PMC6368854

[8] Compston A Coles A. Multiple sclerosis. Lancet (2008) 372:1502–17. 10.1016/S0140-6736(08)61620-7 18970977

[9] Barnes KA Cohen AL Power JD Nelson SM Dosenbach YBL Miezin FM Identifying basal ganglia divisions in individuals using resting-state functional connectivity MRI. Front Syst Neurosci (2010) 4:18 10.3389/fnsys.2010.00018 20589235 PMC2892946

[10] Tortorella C Romano R Direnzo V Taurisano P Zoccolella S Iaffaldano P Load-dependent dysfunction of the putamen during attentional processing in patients with clinically isolated syndrome suggestive of multiple sclerosis. Mult Scler (2013) 19(9):1153–60. 10.1177/1352458512473671 23329700

[11] Sbardella E Petsas N Tona F Pantano P. Resting-state fMRI in MS: general concepts and brief overview of its application. Biomed Res Int (2015) 2015:212693. 10.1155/2015/212693 26413509 PMC4564590

[12] Fling BW Gera Dutta G Horak FB Functional connectivity underlying postural motor adaptation in people with multiple sclerosis. Neuroimage Clin (2015) 8:281–9. 10.1016/j.nicl.2015.04.023 26106552 PMC4474363

[13] Dogonowski AM Siebner HR Soelberg SP Paulson OB Dyrby TB Blinkenberg M Resting-state connectivity of pre-motor cortex reflects disability in multiple sclerosis. Acta Neurol Scand (2013) 128:328–35. 10.1111/ane.12121 23461607

[14] Dogonowski AM Siebner HR Sorensen PS Wu X Biswal B Paulson OB Expanded functional coupling of subcortical nuclei with the motor resting-state network in multiple sclerosis. Mult Scler (2013) 19(5):559–66. 10.1177/1352458512460416 23012251

[15] Finke C Schlichting J Papazoglou S Scheel M Freing A Soemmer C Altered basal ganglia functional connectivity in multiple sclerosis patients with fatigue. Mult Scler (2014) 21(7):925–34. 10.1177/1352458514555784 25392321

[16] Di Martino A Scheres A Margulies DS Kelly AMC Uddin LQ Shehzad Z Functional connectivity of human striatum: a resting state fMRI study. Cereb Cortex (2008) 18(12):2735–47. 10.1093/cercor/bhn041 18400794

[17] Jung WH Jang JH Park JW Kim E Goo EH Im OS Unravelling the intrinsic functional organization of the human striatum: a parcellation and connectivity study based on resting-state fMRI. PLoS One (2014) 9(9):e106768. 10.1371/journal.pone.0106768 25203441 PMC4159235

[18] Felger JC Li Z Haroon E Woolwine BJ Jung MY Hu X Inflammation is associated with decreased functional connectivity within corticostriatal reward circuitry in depression. Mol Psychiatry (2016) 21(10):1358–65. 10.1038/mp.2015.168 26552591 PMC4862934

[19] Wang Z Wang X Liu J Chen J Liu X Nie G Acupuncture treatment modulates the corticostriatal reward circuitry in major depressive disorder. J Psychiatr Res (2017) 84:18–26. 10.1016/j.jpsychires.2016.09.014 27693978 PMC5125902

[20] Di Martino A Kelly C Grzadzinski R Zuo XN Mennes M Mairena MA Aberrant striatal functional connectivity in children with autism. Biol Psychiatry (2011) 69(9):847–56. 10.1016/j.biopsych.2010.10.029 21195388 PMC3091619

[21] Harrison BJ Soriano-Mas C Pujol J Ortiz H López-Solà M Hernández-Ribas R Altered corticostriatal functional connectivity in obsessive-compulsive disorder. Arch Gen Psychiatry (2009) 66(11):1189–200. 10.1001/archgenpsychiatry.2009.152 19884607

[22] Polman CH Reingold SC Banwell B Clanet M Cohen JA Filippi M Diagnostic criteria for multiple sclerosis: 2010 revisions to the McDonald criteria. Ann Neurol (2011) 69(2):292–302. 10.1002/ana.22366 21387374 PMC3084507

[23] Oldfield RC The assessment and analysis of handedness: the Edinburgh inventory. Neuropsychologia (1971) 9(1):97–113. 10.1016/0028-3932(71)90067-4 5146491

[24] Kurtzke JF Rating neurologic impairment in multiple sclerosis: an expanded disability status scale (EDSS). Neurology (1983) 33(11):1444–52. 10.1212/WNL.33.11.1444 6685237

[25] Maldjian JA Laurienti PJ Kraft RA Burdette JH An automated method for neuroanatomic and cytoarchitectonic atlas-based interrogation of fMRI data sets. Neuroimage (2003) 19:1233–9. 10.1016/S1053-8119(03)00169-1 12880848

[26] Whitfield-Gabrieli S Nieto-Castanon A. *Conn*: a functional connectivity toolbox for correlated and anticorrelated brain networks. Brain Connect (2012) 2(3):125–41. 10.1089/brain.2012.0073 22642651

[27] Sbardella E Tona F Petsas N Upadhyay N Piattella M Filippini N Functional connectivity changes and their relationship with clinical disability and white matter integrity in patients with relapsing-remitting multiple sclerosis. Mult Scler (2015) 21(13):1681–93. 10.1177/1352458514568826 26041799

[28] Janssen AL Boster A Patterson BA Abduljalil A Prakash RS. Resting-state functional connectivity in multiple sclerosis: an examination of group differences and individual differences. Neuropsychologia (2013) 51(13):2918–29. 10.1016/j.neuropsychologia.2013.08.010 23973635

[29] Doyon J Bellec P Amsel R Penhune V Monchi O Carrier J Contributions of the basal ganglia and functionally related brain structures to motor learning. Behav Brain Res (2009) 199:61–75. 10.1016/j.bbr.2008.11.012 19061920

[30] Alexander GE Crutcher MD Neural representations of the target (goal) of visually guided arm movements in three motor areas of the monkey. J Neurophysiol (1990) 64(1):164–78. 2388063 10.1152/jn.1990.64.1.164

[31] Alexander GE DeLong MR Strick PL Parallel organization of functionally segregated circuits linking basal ganglia and cortex. Annu Rev Neurosci (1986) 9:357–81. 10.1146/annurev.neuro.9.1.357 3085570

[32] Cui LB Liu K Li C Wang LX Guo F Tian P Putamen-related regional and network functional deficits in first-episode schizophrenia with auditory verbal hallucinations. Schizophr Res (2016) 173:13–22. 10.1016/j.schres.2016.02.039 26995674

[33] Dietrich A Hollmann M Mathar D Villringer A Horstmann A Brain regulation of food craving: relationships with weight status & eating behavior. Int J Obes (Lond) (2016) 40(6):982–9. 10.1038/ijo.2016.28 26883294

[34] Postuma RB Dagher A. Basal ganglia functional connectivity based on a meta-analysis of 126 positron emission tomography and functional magnetic resonance imaging publications. Cereb Cortex (2006) 16(10):1508–21. 10.1093/cercor/bhj088 16373457

[35] Karim HT Huppert TJ Erickson KI Wollam ME Sparto PJ Sejdić E Motor sequence learning-induced neural efficiency in functional brain connectivity. Behav Brain Res (2017) 319:87–95. 10.1016/j.bbr.2016.11.021 27845228 PMC5183470

[36] Calabrese M Rinaldi F Grossi P Mattisi I Bernardi V Favaretto A Basal ganglia and frontal/parietal cortical atrophy is associated with fatigue in relapsing-remitting multiple sclerosis. Mult Scler (2010) 16(10):1220–8. 10.1177/1352458510376405 20670981

[37] Curtis CE D’Esposito M. Persistent activity in the prefrontal cortex during working memory. Trends Cogn Sci (2003) 7(9):415–23. 10.1016/S1364-6613(03)00197-9 12963473

[38] Turner MP Hubbard NA Himes LM Faghihahmadabadi S Hutchison JL Bennett IJ Cognitive slowing in Gulf War Illness predicts executive network hyperconnectivity: study in a population-representative sample. Neuroimage Clin (2016) 12:535–41. 10.1016/j.nicl.2016.08.022 27672557 PMC5030369

[39] Disbrow EA Sigvardt KA Franz EA Turner RS Russo KA Hinkley LB Movement activation and inhibition in Parkinson’s disease: a functional imaging study. J Parkinsons Dis (2013) 3(2):181–92. 10.3233/JPD-130181 23938347 PMC4586119

[40] Pochon J-BB Levy R Poline JB Crozier S Lehéricy S Pillon B The role of dorsolateral prefrontal cortex in the preparation of forthcoming actions: an fMRI study. Cereb Cortex (2001) 11(3):260–6. 10.1093/cercor/11.3.260 11230097

[41] Hoshi E Tanji J. Distinctions between dorsal and ventral premotor areas: anatomical connectivity and functional properties. Curr Opin Neurobiol (2007) 17(2):234–42. 10.1016/j.conb.2007.02.003 17317152

[42] Hubbard NA Hutchison JL Turner MP Sundaram S Robinson D Strain J Asynchrony in executive networks predicts cognitive slowing in multiple sclerosis. Neuropsychology (2016) 30(1):75–86. 10.1037/neu0000202 26146853

[43] Hubbard NA Hutchison JL Motes MA Shokri-Kojori E Bennett IJ Brigante RM Central executive dysfunction and deferred prefrontal processing in veterans with Gulf War illness. Clin Psychol Sci (2014) 2(3):319–27. 10.1177/2167702613506580 25767746 PMC4352953

[44] Mikell CB Banks GP Frey HP Youngerman BE Nelp TB Karas PJ Frontal networks associated with command following after hemorrhagic stroke. Stroke (2015) 46:49–57. 10.1161/STROKEAHA.114.007645 25492905

[45] Ora H Wada M Salat D Kansaku K Arm crossing updates brain functional connectivity of the left posterior parietal cortex. Sci Rep (2016) 6:28105 10.1038/srep28105 27302746 PMC4908406

[46] Desbordes G Li A Loggia ML Kim J Schalock PC Lerner E Evoked itch perception is associated with changes in functional brain connectivity. Neuroimage Clin (2015) 7:213–21. 10.1016/j.nicl.2014.12.002 25610783 PMC4300003

[47] Schmahmann JD Pandya DN. Anatomical investigation of projections to the basis pontis from posterior parietal association cortices in rhesus monkey. J Comp Neurol (1989) 289(1):53–73. 10.1002/cne.902890105 2478597

[48] Vahdat S Darainy M Milner TE Ostry DJ. Functionally specific changes in resting-state sensorimotor networks after motor learning. J Neurosci (2011) 31(47):16907–15. 10.1523/JNEUROSCI.2737-11.2011 22114261 PMC3260885

[49] Prosperini L Piattella MC Giannni C Pantano P. Functional and structural brain plasticity enhanced by motor and cognitive rehabilitation in multiple sclerosis. Neural Plast (2015) 2015:481574. 10.1155/2015/481574 26064692 PMC4438192

[50] Filippi M Agosta F Spinelli EG Rocca MA. Imaging resting state brain function in multiple sclerosis. J Neurol (2013) 260:1709–13. 10.1007/s00415-012-6695-z 23052604

[51] Pantano P Iannetti GD Caramia F Mainero C Di Legge S Bozzao L Cortical motor reorganization after a single clinical attack of multiple sclerosis. Brain (2002) 125:1607–15. 10.1093/brain/awf164 12077009

[52] Zeller D Classen J Plasticity of the motor system in multiple sclerosis. Neuroscience (2014) 283:222–30. 10.1016/j.neuroscience.2014.05.043 24881573

[53] Fransson P Marrelec G. The precuneus/posterior cingulate cortex plays a pivotal role in the default mode network: evidence from a partial correlation network analysis. Neuroimage (2008) 42:1178–84. 10.1016/j.neuroimage.2008.05.059 18598773

[54] Raichle ME MacLeod AM Snyder AZ Powers WJ Gusnard DA Shulman GL. A default mode of brain function. Proc Natl Acad Sci U S A (2001) 98(2):676–82. 10.1073/pnas.98.2.676 11209064 PMC14647

[55] Leech R Sharp DJ Addis D Wong A Schacter D Alsaadi T The role of the posterior cingulate cortex in cognition and disease. Brain (2014) 137:12–32. 10.1093/brain/awt162 23869106 PMC3891440

[56] Hwang JW Xin SC Ou YM Zhang WY Liang YL Chen J Enhanced default mode network connectivity with ventral striatum in subthreshold depression individuals. J Psychiatr Res (2016) 76:111–20. 10.1016/j.jpsychires.2016.02.005 26922247 PMC4838997

[57] Treserras S Boulanouar K Conchou F Simonetta-Moreau M Berry I Celsis P Transition from rest to movement: brain correlates revealed by functional connectivity. Neuroimage (2009) 48:207–16. 10.1016/j.neuroimage.2009.06.016 19527788

[58] Zhang Y Li K Ren Y Cui F Xie Z Shin JY Acupuncture modulates the functional connectivity of the default mode network in stroke patients. Evid Based Complement Alternat Med (2014) 2014:765413. 10.1155/2014/765413 24734113 PMC3963376

[59] Hu S Li CSR. Neural processes of preparatory control for stop signal inhibition. Hum Brain Mapp (2012) 33(12):2785–96. 10.1002/hbm.21399 21976392 PMC3293936

[60] Pantano P Petsas N Tona F Sbardella E The role of fMRI to assess plasticity of the motor system in MS. Front Neurol (2015) 6:55 10.3389/fneur.2015.00055 25852634 PMC4360702

